# Physiological and Molecular Mechanisms of Tree Responses to Combined Drought and Low-Nitrogen Stress: A Review

**DOI:** 10.3390/ijms27198853

**Published:** 2026-10-04

**Authors:** Yang Xu, Zhao Yang, Lijing Yu, Xia Wang, Chenghao Li, Ming Wei

**Affiliations:** 1 State Key Laboratory of Tree Genetics and Breeding, Northeast Forestry University, Harbin 150040, China; 15148962246@163.com (Y.X.); 15822929677@163.com (Z.Y.); ylj1140602651@163.com (L.Y.); 13789644342@163.com (X.W.); 2 School of Forestry, Northeast Forestry University, Harbin 150040, China

**Keywords:** drought stress, low-nitrogen stress, combined stress, ABA signaling, carbon–nitrogen metabolism, forest trees

## Abstract

Drought and low nitrogen often co-occur in forests, yet their combined effects cannot be inferred reliably from single-stress studies. This review synthesizes physiological and molecular evidence for tree responses to combined drought and low nitrogen at four levels, namely stress perception, signal transduction, metabolic reprogramming, and adaptive phenotypes. It compares single-stress baselines and evaluates reported interactions in photosynthesis, water-use efficiency, root traits, and carbon–nitrogen allocation. The review distinguishes direct tree evidence from mechanistic context derived from crops and model plants. Abscisic acid–nitrogen signaling crosstalk is considered alongside reactive oxygen species, energy, hydraulics, and transcriptional regulation as candidate integration routes. Candidate genes are classified by species context and experimental evidence; responsiveness to the two single stresses is not interpreted as proof of a combined-stress function. The review also assesses the opportunities and current limitations of multi-omics and genome editing for trees. It identifies priorities for factorial experiments, multi-year validation, and molecular breeding for climate-resilient forestry.

## 1. Introduction

Global climate change is increasing the frequency and intensity of drought while reducing nitrogen availability, making these two stresses critical constraints on sustainable forestry [1,2,3]. Drought and nitrogen deficiency often co-occur in natural ecosystems, resulting in combined stress with complex interactive effects [1,3]. A systematic review of combined drought and salinity stress demonstrated that plants activate distinct mechanisms under combined stress that cannot be predicted from responses to individual stress alone [4,5]. Because tree varieties selected for tolerance to a single stress often underperform under field conditions, elucidating the unique responses and key regulatory genes involved in combined stress is essential for breeding trees capable of sustaining productivity under variable environmental conditions.

Trees differ from annual model plants in several characteristics that are relevant to stress adaptation. Their long generation cycles and perennial growth can result in cumulative physiological effects that differ from those observed during short-term stress responses [6,7]. Seasonal source–sink dynamics regulate carbon and nitrogen allocation, while extensive root systems can buffer acute stress but may impose substantial metabolic costs under chronic stress conditions [6,7,8]. Consequently, knowledge of single-stress responses derived from model plants cannot fully explain how trees adapt to combined stresses.

To systematically synthesize this field, this review is structured around stress perception, signal transduction, metabolic reprogramming, and adaptive phenotypes, with single-stress responses serving as a baseline for evaluating interactive effects. Crosstalk between abscisic acid (ABA) signaling and nitrogen transport is discussed as a potential point of integration, alongside the roles of reactive oxygen species (ROS), energy status, hydraulics, and transcriptional regulation [3,9,10]. By integrating recent advances in multi-omics and clustered regularly interspaced short palindromic repeats/CRISPR-associated protein 9 (CRISPR/Cas9) technologies [11,12,13], this review establishes a unified framework for understanding combined drought and nitrogen stress responses in trees and extends beyond previous reviews of drought responses in woody crops [14].

## 2. Tree-Specific Context for Drought and Low-Nitrogen Adaptation

Trees experience drought and nitrogen limitations within perennial, spatially heterogeneous, and developmentally structured systems. Their long-life cycles allow stress effects to accumulate across seasons, while deep and heterogeneous root systems, secondary growth, hydraulic architecture, and seasonal source–sink dynamics link local stress perception to whole-plant resource allocation. Consequently, evidence from annual crops and model plants is valuable for generating mechanistic hypotheses but should not be regarded as direct functional evidence for forest trees. In addition, tree species and genotypes vary considerably in their responses and performance under drought and low-nitrogen conditions. Key axes of variation include stomatal regulation, xylem embolism resistance, osmotic adjustment, fine-root proliferation, nitrogen uptake preference, and the capacity to maintain nonstructural carbon reserves. These traits should be evaluated as continuous, context-dependent strategies rather than as fixed categories of tolerance or sensitivity. Recent studies in trees have shown that nitrogen-efficient poplar genotypes differ in fine-root plasticity, while hydraulic and stomatal strategies vary both among and within tree species [15,16].

A systematic understanding of combined stress requires clear baselines for each individual stress. Drought and nitrogen limitation affect tree growth through overlapping, multiscale processes of stress perception and signaling. ABA is a major drought-responsive hormone that coordinates stomatal and transcriptional responses, while hydraulic signals, osmotic status, calcium, ROS, and peptide signals also contribute to drought responses. Nitrate transporter 1.1 (NRT1.1), also known as nitrate transporter 1/peptide transporter family 6.3 (NPF6.3), functions as a nitrate transceptor in some plant systems. Responses to low nitrogen additionally involve the sensing of external and internal nitrate status, high-affinity nitrate transporter 2 (NRT2) systems, ammonium transporters (AMTs), carbon-to-nitrogen (C/N) metabolites, and transcriptional regulators. We therefore use crosstalk between ABA signaling and nitrogen transport as an illustrative integration point rather than as the sole or universal module for stress perception. ROS signaling, energy status, osmotic adjustment, and carbon–nitrogen balance represent additional points of convergence through which drought and nitrogen limitation may interact.

### 2.1. Photosynthetic Inhibition and Osmotic Adjustment Under Drought Stress

Drought stress rapidly reduces photosynthetic rates in trees, and this response undergoes a stage-dependent transition. Under mild drought, stomatal closure is the predominant limitation on photosynthesis. ABA activates anion channels in guard cells, promoting potassium and anion efflux, guard cell water loss, and subsequent stomatal closure [2,3]. As drought severity increases, non-stomatal limitations become progressively more pronounced, including reduced photosystem II (PSII) photochemical efficiency, decreased ribulose-1,5-bisphosphate carboxylase/oxygenase (Rubisco) activity, and degradation of photosynthetic pigments [17]. The threshold at which this transition occurs varies markedly among species. For example, Scots pine (*Pinus sylvestris*) maintains relatively high photosynthetic rates under prolonged drought through sustained osmotic adjustment and efficient water uptake [8], whereas poplar exhibits pronounced non-stomatal limitations even under moderate drought conditions [17,18].

Osmotic adjustment is another key strategy for coping with drought stress. Drought induces the accumulation of compatible osmolytes, such as proline, soluble sugars, and glycine betaine, which lower cellular osmotic potential and help maintain turgor and water uptake [2]. Proline accumulation is commonly associated with increased expression of Δ^1^-pyrroline-5-carboxylate synthetase (P5CS) and reduced expression of proline dehydrogenase (ProDH), although the magnitude and direction of these changes vary among species and experimental conditions [3]. P5CS catalyzes the first committed steps in proline biosynthesis from glutamate, and its expression can be regulated through both ABA-dependent and ABA-independent pathways [19]. Notably, osmotic adjustment strategies vary among tree species. Poplar relies mainly on soluble sugar accumulation, whereas pine depends more on the coordinated accumulation of proline and glycine betaine [8]. In addition to osmotic regulation, drought stress activates antioxidant defense systems that mitigate oxidative damage caused by ROS [2,20]. These antioxidant systems, comprising both enzymatic and non-enzymatic components, play a central role in drought tolerance by collectively maintaining redox homeostasis under water-deficit conditions [21].

### 2.2. Nitrogen Uptake and Metabolic Reprogramming Under Low-Nitrogen Stress

Under low-nitrogen stress, plants enhance nitrogen uptake and assimilation efficiency by increasing root surface area and regulating the activities of key nitrogen-metabolizing enzymes [22,23]. Trees activate high-affinity nitrate transport systems; for example, members of the high-affinity nitrate transporter 2 (NRT2) family, particularly *NRT2.1*, *NRT2.4*, and *NRT2.5*, are strongly upregulated under low-nitrogen conditions [24]. For ammonium uptake, AMT1 family transporters exhibit species-specific responses. Poplar upregulates *AMT1.1* expression under low nitrogen, whereas pine tends to enhance nitrate uptake instead [17]. In *Malus prunifolia*, drought stress significantly affects both ammonium and nitrate uptake, with AMT genes showing greater sensitivity to drought than NRT genes [25].

The nitrogen assimilation machinery undergoes substantial adjustments under low-nitrogen stress. Nitrate reductase (NR) and nitrite reductase (NiR) activities decline in response to reduced substrate availability, whereas the GS1 isoform of glutamine synthetase (GS) maintains relatively high expression levels in roots [17]. Concurrently, nitrogen remobilization from senescing leaves to developing tissues is enhanced through increased phloem transport of amino acids, particularly glutamine and asparagine [26], representing an important strategy for improving nitrogen use efficiency under low-nitrogen conditions. In banana seedlings, nitrogen deficiency was associated with differential expression of myeloblastosis (MYB) and GAI, RGA, and SCR (GRAS) family transcription factors (TFs), as well as changes in root-system development [27]. Studies of nitrogen supplementation under drought stress also reveal context-dependent effects in trees. For example, transcriptome analysis of *Sophora japonica* roots exposed to polyethylene glycol (PEG)-induced drought stress under different nitrogen conditions showed that nitrogen starvation resulted in more downregulated genes than normal nitrogen conditions, particularly those involved in carbon metabolism [28]. In *Abies fabri* seedlings, nitrogen supply significantly altered ecophysiological responses to drought stress, including photosynthetic parameters and water-use efficiency [29]. Table 1 provides a systematic comparison of tree responses to drought and low-nitrogen stress across multiple dimensions.

## 3. Interactive Effects of Combined Drought and Low-Nitrogen Stress

Drought and low nitrogen often co-occur, but their interaction should be distinguished from studies examining nitrogen supplementation under drought. The term “combined drought and low nitrogen” refers to factorial experiments that include drought, low-nitrogen, and combined drought and low-nitrogen treatments, whereas “drought with nitrogen supplementation” refers to studies assessing whether supplemental nitrogen modifies drought responses without directly addressing tolerance to low nitrogen. Interaction effects should be evaluated using factorial models that explicitly test the drought × nitrogen interaction. On a response scale defined a priori, an additive response is expected when the combined effect equals the sum of the effects of the two individual stresses. A synergistic response occurs when the combined effect deviates from the additive expectation in the same direction and is stronger than predicted by the individual stress effects, whereas an antagonistic response occurs when the combined effect is weaker than expected or shifts toward the direction of the individual effects. Cross-system evidence from multiple studies [30,31,32] indicates that additive, antagonistic, and synergistic interactions depend on the response trait and experimental context. Without an appropriate factorial interaction test and corresponding controls, descriptive comparisons alone cannot establish the presence of synergy or antagonism.

The strength of evidence also varies among species. Direct evidence from trees includes factorial physiological, transcriptomic, and functional studies conducted in woody species. Recent studies of *S. japonica* and four urban tree species illustrate the value of explicitly manipulating water and nitrogen availability when assessing transcriptional and nitrogen-uptake responses [28,33]. Evidence from *Arabidopsis* and crop species is discussed as mechanistic context, with its origin indicated. For example, the dynamic interaction reported by Xue et al. in *Arabidopsis* illustrates a model-plant response [30], whereas the transcriptional reprogramming observed in *Camellia oleifera* following nitrogen supplementation under drought addresses the effects of nitrogen supplementation rather than combined drought and low-nitrogen stress [31].

### 3.1. Impairment of Photosynthetic Carbon Assimilation

Drought limits CO_2_ diffusion through stomatal closure, whereas low nitrogen limits photosynthetic carboxylation capacity through reduced Rubisco content. These mechanisms can result in substantial combined inhibition of photosynthetic carbon assimilation; however, the interaction should be characterized as additive or synergistic only when supported by an appropriate factorial analysis. In barley, the combined treatment caused greater downregulation of photosynthetic proteins than either individual treatment, providing crop-based evidence of a non-additive response [34]. In poplar, drought and low nitrogen have been associated with reduced photosynthesis and altered nitrogen uptake, but the magnitude and direction of their interaction depend on genotype, treatment intensity, and duration [24]. Similarly, nitrogen supplementation studies in *Alhagi sparsifolia* should be interpreted as evidence that nitrogen availability modulates drought responses rather than as evidence of adaptation to combined drought and low-nitrogen stress [35].

### 3.2. Divergent Responses of Water Use Efficiency and Nutrient Uptake

Changes in water-use efficiency (WUE) under combined stress exhibit species-specific patterns. Under moderate drought, reduced nutrient uptake, together with changes in leaf nitrogen status, chlorophyll content, stomatal conductance, and transpiration, can alter instantaneous WUE (WUEi) [17,29,36]. The magnitude and direction of WUEi responses should be evaluated using species-specific factorial experiments in trees. In *Calligonum mongolicum* seedlings, nitrogen supplementation enhanced leaf–root coordination of hormonal, redox, and carbohydrate metabolism and improved drought tolerance [37]. These findings illustrate how nutrient supply can modify drought responses but do not establish responses to simultaneous drought and low-nitrogen stress.

The ABA signaling pathway coordinates plant responses to multiple stresses [38,39]. Drought-induced ABA signaling can influence root development and nitrogen transport processes, including the regulation of NRT1.1/NPF6.3 in model systems [10]. These observations identify potential molecular links between water and nitrogen signaling pathways. However, their contributions to responses to simultaneous drought and low-nitrogen stress in trees require direct validation using factorial experiments.

With respect to nutrient uptake, drought-induced inhibition of soil nitrogen mineralization, together with reduced root nitrogen uptake under low-nitrogen stress, can produce additive effects. Reduced soil moisture further limits the mass flow and diffusion of nitrogen, ultimately resulting in significantly decreased root uptake fluxes of NO_3_^−^ and NH_4_^+^ [40]. A ^13^C isotope tracer experiment in *Sophora moorcroftiana* demonstrated that, compared with single-stress treatments, combined drought and nitrogen deficiency significantly reduced ^13^C accumulation in plants while increasing the proportion of residual ^13^C in soil, suggesting altered carbon allocation from plants to soil microorganisms under combined stress [40]. In guar (*Cyamopsis tetragonoloba*), drought-induced changes in enzymes involved in carbon and nitrogen metabolism were accompanied by increased carbohydrate accumulation, highlighting the coordinated interaction between carbon and nitrogen metabolism in drought tolerance [41].

### 3.3. Root Architecture and Functional Plasticity Trade-Offs

**Figure 1:**
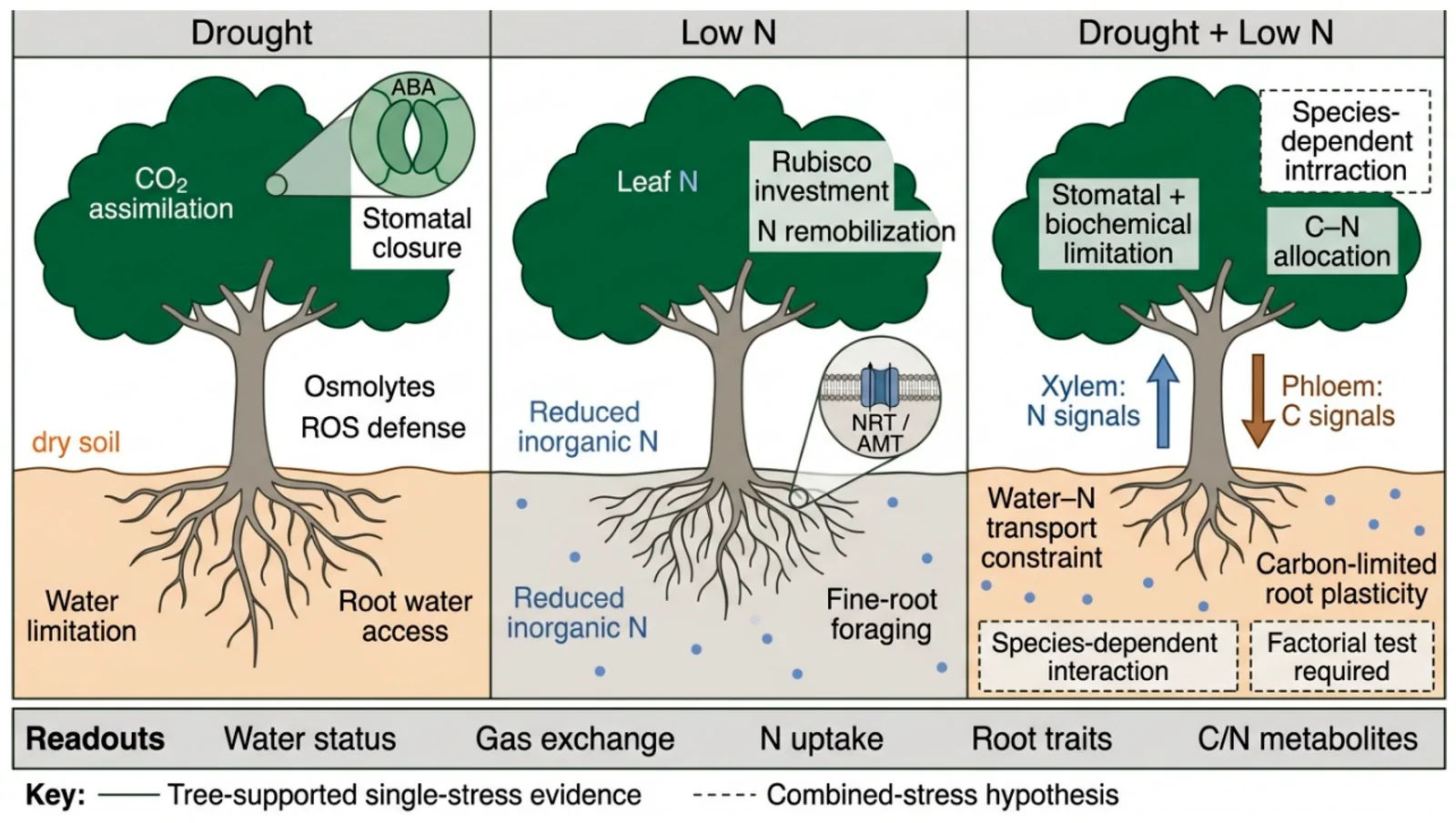
A multi-level comparison of tree responses to drought, low nitrogen, and their combined treatment. The panels integrate soil resource limitation, root traits and nitrogen acquisition, canopy gas exchange, osmotic and redox adjustment, and root–shoot carbon–nitrogen signaling. Solid outlines indicate tree-supported single-stress evidence; dashed outlines identify candidate combined-stress interactions that require species-specific factorial validation. The lower strip lists the complementary physiological and metabolic readouts for factorial drought × nitrogen experiments.

Tree root architecture exhibits substantial plasticity under water and nitrogen limitation; however, the direction of biomass allocation under concurrent drought and low-nitrogen availability depends on species, developmental stage, carbon status, and treatment duration. In Scots pine, prolonged drought alters carbon allocation to roots [8], whereas studies in poplar demonstrate that nitrogen availability modulates drought-associated root development [17]. In *Catalpa bungei*, factorial drought and nitrogen treatments altered proline accumulation, antioxidant activity, root development, and biomass allocation [42]. Collectively, these studies identify root allocation and function as key dimensions of the response to combined stress. To date, consistent effect sizes and a universal trade-off under combined stress have not been established across tree species. Consequently, Figure 1 summarizes testable response domains rather than uniform directional outcomes.

Arbuscular mycorrhizal fungi (AMF) can modulate host water relations and nutrient acquisition under water-limited conditions. By extending the soil exploration volume of roots, AMF hyphal networks can enhance nitrogen acquisition; in sugarcane, this effect has been associated with improved host plant water status under drought conditions [43]. In a factorial greenhouse experiment with *Commiphora myrrha* seedlings, mycorrhizal plants exhibited greater root and shoot biomass and higher nutrient concentrations than non-mycorrhizal controls, while their drought response index increased under lower water availability [44]. Nevertheless, the contribution of AMF to tree performance under combined drought and low-nitrogen stress remains insufficiently resolved because of limited direct factorial evidence. Consequently, rhizosphere management represents a promising research direction, requiring tree-focused experiments that simultaneously quantify AMF colonization, plant water status, nitrogen acquisition, and growth under combined-stress regimes.

## 4. Molecular Regulatory Networks Under Combined Stress

Combined drought and low-nitrogen stress may involve crosstalk among ABA and nitrogen signaling pathways, as well as redox, energy, hydraulic, and transcriptional processes. In forest trees, direct evidence for a single hierarchical regulatory network integrating ABA and nitrogen signaling remains limited. NAM, ATAF, and CUC (NAC) TF families are discussed here as candidate integrators because they are represented in drought- and nitrogen-responsive datasets; however, their functions under combined stress require direct validation using factorial experiments. Evidence from crops and model plants is used to identify testable mechanisms rather than to establish regulatory pathways in forest trees [5,10,45]. A review of drought-resistance engineering in forest trees identifies osmotic adjustment, antioxidant defense, and WUE as potential targets, but findings from laboratory studies require validation across tree genotypes, developmental stages, and field-relevant environments [46].

### 4.1. Crosstalk Between ABA Signaling and Nitrogen Signaling

The core components of the ABA signaling pathway include pyrabactin resistance/pyrabactin resistance-like/regulatory component of ABA receptor (PYR/PYL/RCAR) proteins, protein phosphatase 2C (PP2C) phosphatases, and SNF1-related protein kinase 2 (SnRK2) kinases. Under drought stress, ABA binds to PYR/PYL receptors, relieving PP2C-mediated inhibition of SnRK2 and thereby enabling downstream stress-responsive transcription [3]. Under combined stress, crosstalk between ABA and nitrogen signaling pathways can occur at multiple levels. For example, SnRK2 kinases can directly phosphorylate the NRT2.1 transporter, thereby affecting its plasma membrane localization and transport activity [19]. On the other hand, nitrate signaling regulates the expression of the key ABA biosynthesis enzyme 9-cis-epoxycarotenoid dioxygenase 3 (NCED3) through NIN-like protein (NLP) TFs, thereby influencing endogenous ABA levels [17]. These two regulatory pathways—ABA-to-NRT signaling, which regulates nitrogen uptake, and nitrate-to-ABA signaling, which influences stomatal regulation—may form a complementary regulatory circuit [5]. In poplar, exogenous nitrogen supply under drought stress promotes the expression of key genes involved in ABA signaling, which may enhance stomatal regulatory capacity [17]. This ABA-nitrogen crosstalk may integrate water and nutrient signals, enabling trees to coordinate carbon and nitrogen acquisition under stress. Recent findings further suggest that SNF1-related protein kinase 1 (SnRK1), a central energy sensor, integrates metabolic and stress signals. By phosphorylating antioxidant enzymes, ion channels, and immune-related factors, SnRK1 may contribute to the reprogramming of carbon–nitrogen metabolism and resource allocation during drought [47].

### 4.2. Transcription Factor Regulatory Networks Integrating Drought and Nitrogen Signals

#### 4.2.1. Evidence Framework and NAC Family as Dual Integrators

TFs are plausible integrators of combined-stress responses, but the evidence should be distinguished by species and level of validation. Throughout this section, we organize the evidence into three categories. Category I comprises direct functional evidence from a tree species for one relevant stress. Category II includes dual-function evidence derived from separate drought and nitrogen experiments involving the same tree gene or ortholog. Category III encompasses mechanistic evidence from *Arabidopsis* or crop species that provides testable hypotheses for trees. In *Populus davidiana*, drought transcriptomic analysis identified APETALA2/ethylene-responsive factor (AP2/ERF), WRKY, C2H2, and NAC TFs among the differentially expressed genes (DEGs), providing tree-specific evidence for candidate regulators under single-stress conditions [19]. Direct functional validation of drought- and nitrogen-responsive regulatory modules under factorial combined-stress conditions remains rare in forest trees. Accordingly, examples from crops and model plants are retained to illustrate candidate mechanisms, whereas conclusions regarding forest-tree breeding are limited to genes supported by tree-specific evidence and are explicitly framed as priorities for further validation.

NAC TFs are increasingly recognized as regulatory nodes that link adaptation to water deficit with nitrogen acquisition. One of the strongest examples supporting a dual-integrator model is STRESS-RESPONSIVE NAC1 (OsSNAC1) in rice. First characterized as a drought-inducible factor enriched in guard cells, *OsSNAC1* overexpression enhanced drought resistance in field-grown rice, promoted stomatal closure, reduced water loss, and increased the expression of stress-responsive genes without causing apparent yield penalties under the reported conditions [48]. Subsequent studies established a direct role for *OsSNAC1* in nitrogen-related regulation. OsSNAC1 is induced by nitrogen deficiency in both roots and shoots and directly activates the nitrate transporter genes *OsNRT2.1*, *OsNRT2.2*, *OsNRT1.1A*, and *OsNRT1.1B*. Its overexpression increased nitrate accumulation, nitrogen uptake, nitrogen use efficiency, biomass, and grain yield, whereas loss of OsSNAC1 produced the opposite phenotypes [49]. Thus, a single NAC regulator can coordinate a drought-response program that conserves water with a nutrient-acquisition program that enhances nitrate uptake.

This convergence is physiologically plausible because drought and nitrogen limitation jointly constrain root function, carbon allocation, stomatal regulation, and redox homeostasis. Under combined stress, a NAC-centered regulatory network could potentially enhance root nutrient-acquisition capacity while limiting transpirational water loss, thereby helping plants maintain growth when both resources are limited. Available mechanistic studies place SNAC1 upstream of targets involved in guard cell regulation, redox homeostasis, ABA-associated responses, and nitrate uptake, supporting its potential role as an integrator of these pathways rather than as a regulator of independent responses [50]. Network analyses have also identified NAC candidates in modules responsive to nitrogen deficiency, including PtaNAC1 in poplar and OsNAC36 in rice, although their direct targets and roles in drought responses remain poorly resolved [51].

Future research should test this model under factorial drought × low-nitrogen treatments and across different developmental stages. Priority experiments include tissue-resolved chromatin profiling, time-series transcriptomics, nitrate flux measurements, and field trials that simultaneously quantify yield and nitrogen-use efficiency. Such studies will help distinguish NAC factors that independently respond to the two stresses from true dual-signal integrators whose shared target networks coordinate responses to drought and low-nitrogen conditions.

#### 4.2.2. Cooperative Participation of AP2/ERF Families

In addition to NAC factors, the AP2/ERF, WRKY, basic leucine zipper (bZIP), and dehydration-responsive element-binding (DREB) families are relevant candidates for the regulation of combined-stress responses. OsDREB1C, an AP2/ERF A1-subfamily TF, activates the photosynthesis-related gene *OsRBCS3* and the nitrogen-metabolism genes *OsNRT2.4* and *OsNRT1.1B*. By enhancing photosynthetic and nitrogen-use efficiency, OsDREB1C increased yield in transgenic rice [52], providing a representative example of a TF with potential relevance to combined-stress engineering. Similarly, Zhou et al. (2022) reported that the AP2/ERF A5-subfamily TF GmTDN1 functions under combined drought and low-nitrogen stress in wheat, enhancing photosynthetic capacity, osmotic adjustment, and antioxidant metabolism through regulation of drought-responsive genes, including those encoding late embryogenesis abundant proteins, and the nitrate transporter gene *NRT2.5* [53]. These crop studies provide mechanistic context, whereas corresponding functional evidence in forest trees remains limited.

#### 4.2.3. HD-ZIP I and GATA/GNC Families in Trees

The homeodomain leucine zipper I (HD-ZIP I) TF family has emerged as a potential integrator of drought adaptation and nutrient acquisition in poplar. In *P. ussuriensis*, PuHox52 was initially characterized for its role in adventitious root formation and was subsequently shown to be specifically induced in roots by nitrogen deficiency, promoting the coordinated uptake of nitrate, phosphate, and iron [54]. Under nitrogen deficiency, PuHox52 directly targets and upregulates nitrate-related genes (*PuNRT1.1*, *PuNRT2.4*, *PuCLC-b*, *PuNIA2*, *PuNIR1*, and *PuNLP1*), phosphate-responsive genes (*PuPHL1A* and *PuPHL1B*), and the iron transporter gene *PuIRT1*, thereby establishing a regulatory network that coordinates multiple nutrient homeostasis processes under nitrogen-limited conditions [54].

In *P. trichocarpa*, the ortholog PtrHox52 is a direct target of the PtrSWRA-PtrAREB1/2 histone H3 lysine 4 trimethylation (H3K4me3) pentameric complex. This complex is recruited to the promoters of drought-tolerance genes, including *PtrHox2*, *PtrHox46*, and *PtrHox52*, under drought stress, where it deposits H3K4me3 marks to maintain their transcriptional activation. CRISPR/Cas9-mediated disruption of the pentameric complex impaired drought tolerance [55]. Increased expression of PtrHox52 improved both drought tolerance and growth. HD-ZIP I members have also been implicated in reducing drought-induced losses in hydraulic conductivity through alterations in vessel architecture [55]. Together, these findings suggest that the conserved Hox52 function across *Populus* species may contribute to nutrient acquisition under nitrogen deficiency and drought tolerance through chromatin-level regulation, positioning the HD-ZIP I family as a potential regulatory nexus linking water and nitrogen stress responses in trees.

The GATA TF GNC in poplar functions as a regulator of nitrogen-use efficiency. CRISPR/Cas9-mediated knockout of *GNC* reduced nitrogen-use efficiency and nitrate uptake, whereas its overexpression enhanced nitrogen-use efficiency through the upregulation of *NRT2.4b/c* and *NIA1* [56,57]. Notably, GNC also contributes to drought responses through stomatal regulation, making it a potential tree TF with dual functions in coordinating water- and nitrogen-related stress responses [58].

Significant differences exist in the transcriptional regulatory mechanisms underlying stress responses in trees and herbaceous plants. For example, overexpression of maize *ZmDOF1* significantly increases nitrogen assimilation capacity and biomass accumulation under low-nitrogen conditions [59], whereas its overexpression in poplar does not confer a growth advantage, with GS activity and total nitrogen content showing no significant differences from those in wild-type plants [60]. This contrast highlights the distinct regulatory mechanisms underlying stress responses in trees and underscores the limitations of directly extrapolating findings from herbaceous plants to trees under stress conditions.

In summary, the current literature identifies candidate transcriptional links between drought and nitrogen responses rather than a validated hierarchical regulatory network in forest trees. The strongest tree-based candidate is GNC, for which separate studies in poplar provide functional evidence for roles in nitrogen-use efficiency and drought-associated stomatal regulation. PuHox52 and PtrHox52 illustrate complementary nitrogen- and drought-related functions in different *Populus* species, but their roles under combined stress remain to be experimentally tested. Regulators identified in crops and model plants are included as hypothesis-generating examples only. Table 2 distinguishes these evidence categories.

### 4.3. Molecular Synergy Between Antioxidant Systems and Osmotic Adjustment

Combined stress can increase the production of ROS, including superoxide anions, hydrogen peroxide, and hydroxyl radicals. ROS function as signaling molecules, but their excessive accumulation can cause lipid peroxidation and protein damage [20,21]. Accordingly, the expression and activity of superoxide dismutase (SOD), catalase (CAT), ascorbate peroxidase (APX), and other antioxidant systems represent important response variables in factorial experiments examining drought and nitrogen stress. In Chinese fir, nitrogen supply enhanced antioxidant enzyme activities and reduced malondialdehyde accumulation under PEG-induced drought, indicating that nitrogen supplementation can modulate drought-induced oxidative damage [28].

Proline, soluble sugars, and glycine betaine can contribute to osmotic adjustment, but their accumulation and transcriptional regulation vary among species, tissues, and experimental conditions. Direct evidence for the transcriptional regulation of osmolyte biosynthesis under combined drought and low-nitrogen stress remains limited in trees. Therefore, coordinated changes in osmolyte levels should be interpreted as physiological responses that require validation in factorial drought and nitrogen experiments rather than as evidence of a universally established transcriptional program.

## 5. Carbon–Nitrogen Metabolic Reprogramming Under Combined Stress

### 5.1. Restricted Nitrogen Acquisition and Amino-Acid Remobilization Under Water and Nitrogen Co-Limitation

Combined drought and low nitrogen constrain carbon assimilation, nitrogen uptake, and the availability of carbon skeletons for amino acid synthesis, thereby driving the reorganization of carbon–nitrogen metabolism. In *Populus simonii*, transcriptome profiling of fine roots under long-term drought and low-nitrogen conditions identified 4642 DEGs. Most AMT genes were strongly induced; the tissue NH_4_^+^/NO_3_^−^ ratio increased under drought, and the aspartate-derived amino acid pathway emerged as a potentially important component of root drought tolerance [24]. Consistent with these findings, under PEG-simulated drought combined with low (0.01 mM NH_4_NO_3_) or normal nitrogen supply, drought reduced root and leaf NH_4_^+^ and NO_3_^−^ concentrations and suppressed NR and glutamate synthase (GOGAT) activities, whereas *AMT1;2* and *AMT1;6* were upregulated. These responses suggest a shift toward ammonium acquisition when nitrate assimilation is constrained [62]. In the woody legume *Robinia pseudoacacia*, low nitrogen repressed genes involved in nitrate transport and assimilation and reduced photosynthetic pigment and carbohydrate levels, while pathways associated with amino acid metabolism and senescence-mediated nitrogen remobilization were activated in roots [63]. Factorial drought × nitrogen-deficiency proteomics in barley further showed that combined stress reduced photosynthesis through both decreased stomatal conductance and increased photorespiration; preferentially induced proteins involved in energy, carbohydrate, and amino acid metabolism; and enhanced protein degradation, thereby increasing the availability of free amino acids [34]. In oilseed rape, low nitrogen strongly depleted major amino acid pools and attenuated the magnitude, but not the overall pattern, of the drought-induced metabolic response, indicating that nitrogen status modulates the extent of metabolic reprogramming under drought [64]. Research in cotton has shown that prolonged drought can alter the coordination of carbon and nitrogen metabolism between roots and shoots [65]. Together, these studies identify candidate metabolic response domains, whereas the direction and magnitude of metabolic fluxes under simultaneous drought and low-nitrogen stress remain to be resolved across tree species through factorial experiments.

### 5.2. Adaptive Shift of Carbon Metabolism from Growth to Defense

The reprogramming of photosynthetic carbon allocation under combined stress involves the conversion of starch to soluble sugars and changes in nonstructural carbohydrate (NSC) pools. A meta-analysis of 32 tree species showed that drought consistently decreases leaf and root nitrogen concentrations while increasing leaf soluble sugars and root starch, with the soluble sugar-to-nitrogen ratio serving as a sensitive indicator of short-term acclimation [66]. In *Pinus massoniana* seedlings exposed to combined high temperature and drought, the soluble sugar-to-starch ratio increased, and *PmTPS1*, *PmTPS5*, and *PmTPPD* were identified as candidate regulators of trehalose accumulation [67]. A two-year factorial experiment examining drought and nitrogen supplementation in coexisting *Pinus koraiensis* and *Fraxinus mandshurica* saplings revealed contrasting species-specific carbon responses: nitrogen supplementation impaired root development and exacerbated carbon limitation in the drought-treated conifer, whereas the drought-sensitive broadleaf species accumulated NSCs despite reduced biomass [68]. Similarly, in *Quercus mongolica* seedlings grown under a drought gradient combined with nitrogen addition, leaf soluble sugar concentrations and the soluble sugar-to-starch ratio increased with nitrogen supply under well-watered conditions but decreased under severe drought, when carbon allocation shifted from growth toward storage-based defense [69]. Because these studies examined nitrogen supplementation rather than low-nitrogen availability, they provide evidence for drought × nitrogen-supply interactions but do not establish responses to combined drought and low-nitrogen stress.

Carbon skeletons are also redirected toward secondary metabolism, including the biosynthesis of phenolics, terpenoids, and alkaloids, which contribute to antioxidant defense and signaling [70]. In the dioecious tree *Salix rehderiana*, drought and nitrogen availability exerted opposing, sex-specific effects on flavonoid metabolism. Drought reduced flavonoid biosynthesis, whereas nitrogen addition increased it in female plants. Under the combined treatment, nitrogen addition enhanced flavonoid biosynthesis and glycosylation in females but reduced glycosylation in males, accompanied by ABA-mediated stomatal regulation and changes in C/N accumulation [71]. In *Camellia oleifera*, nitrogen supply under drought restored NSC balance and chlorophyll content and maintained flavonoid–sucrose homeostasis through coordinated regulation of genes involved in starch and sucrose metabolism and flavonoid biosynthesis, including *CoCHI*, *CoLAR*, *CoSPS*, and *CoInv* [32]. These woody-plant studies show that water and nitrogen availability jointly influence secondary carbon allocation, although nitrogen-addition experiments alone do not establish an interaction between drought and low-nitrogen stress. Excessive activation of secondary metabolism may further consume carbon skeletons, imposing an additional carbon cost when carbon availability is already limited [72]. This additional carbon demand may contribute to growth inhibition in trees under combined stress.

### 5.3. Reprogramming of Nitrogen Assimilation and Amino Acid Metabolism

Under combined stress, the nitrogen assimilation machinery undergoes substantial remodeling. NR activity declines in response to reduced substrate availability and ABA-mediated regulation, while the activities of the glutamine synthetase/glutamate synthase (GS/GOGAT) cycle are significantly reduced [24]. Nevertheless, sustained expression of the GS1 isoform in roots remains important for maintaining primary nitrogen assimilation. Studies of transgenic poplar expressing pine *GS1a* demonstrate that GS1a contributes to nitrogen homeostasis and reduces ammonium toxicity under drought conditions [61]. In *Camellia oleifera*, nitrogen supplementation under drought restored nonstructural carbohydrate balance and enhanced antioxidant capacity. Furthermore, coexpression network analysis identified two highly interconnected modules enriched in genes associated with carbon metabolism, nitrogen metabolism, and secondary metabolism [31].

Under combined stress, amino acid metabolism may shift from protein synthesis toward the accumulation of proline and γ-aminobutyric acid (GABA). Proline functions as both an osmolyte and a molecular chaperone, contributing to protein stabilization, ROS scavenging, and maintenance of cellular redox balance [24]. GABA accumulation is closely associated with cytoplasmic acidification. Combined stress can decrease cytoplasmic pH, thereby activating glutamate decarboxylase and promoting the conversion of glutamate to GABA [12]. Consistent with these metabolic shifts, transcriptome analysis of *S. japonica* roots exposed to PEG-induced drought stress under varying nitrogen conditions showed that carbon metabolism was most strongly affected by the interaction between drought and nitrogen availability [28].

### 5.4. Long-Distance Root–Shoot Carbon–Nitrogen Signaling

**Figure 2:**
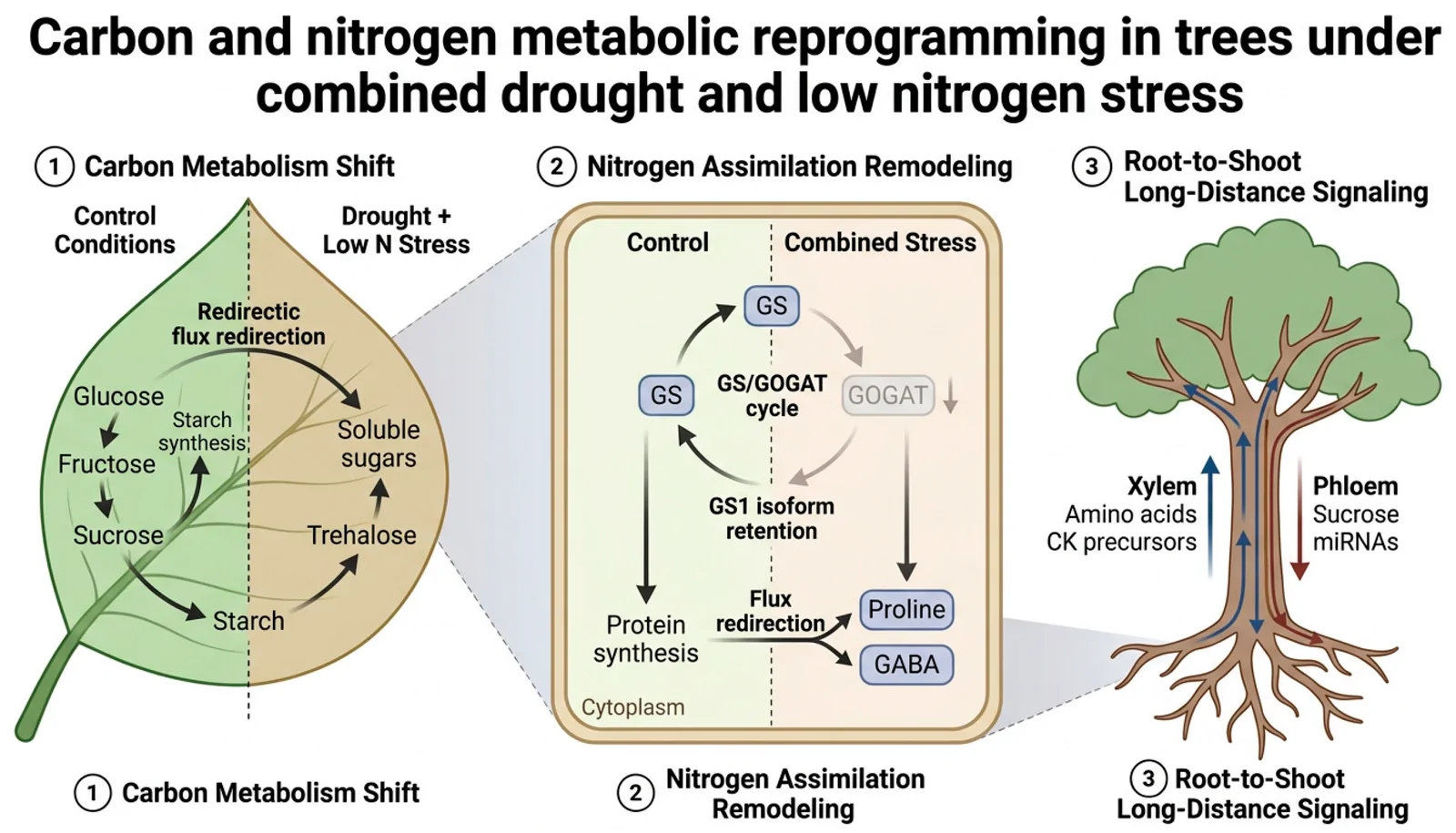
A conceptual model of carbon–nitrogen metabolic reprogramming under drought and low nitrogen. Carbon redistribution, nitrogen assimilation, and xylem–phloem communication are shown as interconnected processes. These connections depict candidate integration routes that require direct testing in tree species under factorial combined stress.

Under combined stress, trees establish a bidirectional root–shoot signaling network in which roots transport amino acids, such as glutamine and asparagine, and cytokinin precursors to the shoots through the xylem to convey nitrogen status, while shoots transport sucrose and microRNA signals to the roots through the phloem to convey carbon status and systemic stress cues [68]. Direct evidence under combined drought and low-nitrogen conditions comes from poplar, where overexpression of *PsAMT1.2* improved growth and photosynthesis under PEG-simulated drought with 0.001 mM NH_4_NO_3_, upregulated genes involved in the GS/GOGAT cycle in both roots and leaves, and enhanced antioxidant defenses. These responses suggest that *PsAMT1.2* contributes to the coordination of nitrogen acquisition with whole-plant carbon–nitrogen balance under combined stress [73]. Supporting studies in woody plants show that xylem-sap amino acid and specialized-metabolite composition respond dynamically to nitrogen availability in *Populus* × *canescens* [74]; stem ABA is associated with hexose mobilization during drought and rewatering in *P. nigra* [75]; nitrogen availability modulates leaf ABA levels and stomatal responses under drought in hybrid poplar [76]; and nitrogen supply enhances leaf–root coordination of hormonal, redox, and carbohydrate metabolism in the desert shrub *C. mongolicum* [37]. Mechanistic studies in herbaceous model plants have identified several mobile signaling components, including the root-derived CLE25 peptide, which promotes ABA biosynthesis in shoots [77]; drought-induced phosphorylation of SWEET sucrose transporters, which enhances phloem sucrose transport to roots [78]; and trehalose-6-phosphate, which coordinates nitrate assimilation with carbon availability [79]. Beyond these model systems, drought alters root–shoot carbon–nitrogen interactions and signal transport in cotton [65], while suppressing nitrogen-metabolism enzymes and promoting compensatory carbohydrate accumulation in *C. tetragonoloba* [41]. As illustrated in Figure 2, carbon–nitrogen metabolic reprogramming in trees under combined stress involves coordinated regulation at three levels: carbon-metabolism reallocation, nitrogen-assimilation remodeling, and root–shoot signal transmission.

## 6. Advances in Multi-Omics Integrative Analysis of Combined Stress Research

### 6.1. Multi-Omics Landscape and Transcriptomic Analysis

Traditional single-factor, single-omics approaches are insufficient to comprehensively characterize the regulatory networks underlying combined stress. In contrast, integrative transcriptomic, proteomic, metabolomic, and epigenomic analyses provide systematic approaches for dissecting tree responses to combined drought and nitrogen limitation [6,13]. In this context, factorial experimental designs are essential for distinguishing individual stress effects from their interactions. A multi-omics study of combined water and nitrogen deficiency across five *Arabidopsis* accessions showed that drought attenuated the nitrogen-deficiency response in a genotype-dependent manner, demonstrating that additive models alone cannot adequately predict interactive drought × low-nitrogen responses [30]. Similarly, an analytical framework that separates additive from interactive molecular responses, developed through transcriptome–metabolome co-analysis of drought and heat stress in *Arabidopsis* [80], provides a useful framework for designing and interpreting tree drought × low-nitrogen experiments.

At the transcriptomic level, RNA sequencing has revealed diverse molecular responses of trees to combined drought and low-nitrogen stress. In fine roots of *P. simonii*, 4642 DEGs were identified under drought and/or low-nitrogen conditions. Drought increased ammonium uptake and the ammonium-to-nitrate ratio, while the aspartate-derived amino acid pathway emerged as a potential contributor to drought tolerance [24]. In the woody legume *R. pseudoacacia*, transcriptomic analysis under combined low-nitrogen and drought conditions showed that low nitrogen repressed genes involved in nitrate transport and assimilation while activating pathways related to amino acid metabolism and nitrogen remobilization in roots; higher nitrogen availability, in contrast, enhanced nitrate metabolism and growth under drought [63]. In *S. japonica* roots, PEG-induced drought under nitrogen-starvation conditions resulted in greater transcriptomic downregulation than either stress alone, with carbon metabolism emerging as the most affected KEGG (Kyoto Encyclopedia of Genes and Genomes) pathway [28]. In *Camellia oleifera*, nitrogen-mediated transcriptional reprogramming coordinated carbon metabolism and flavonoid biosynthesis under drought, while coexpression network analysis identified two interconnected modules enriched in carbon and nitrogen metabolism [31]. Complementary evidence from other combined stresses in trees also points to carbon–nitrogen metabolic reprogramming as a recurring response. For example, heat × drought stress in *P. massoniana* identified starch and sucrose metabolism as a core carbon-metabolism pathway, accompanied by coordinated expression of *PmTPS1*, *PmTPS5*, and *PmTPPD* [67]. Similarly, drought stress in *P. davidiana* enriched DEGs involved in photosynthesis, carbon metabolism, and hormone signaling, including members of the NAC, MYB, bZIP, and AP2/ERF transcription factor families [19]. Nevertheless, molecular signatures specific to combined drought and low-nitrogen stress remain poorly defined in trees.

### 6.2. Proteomic, Metabolomic, and Epigenomic Evidence

Proteomic evidence for combined drought and low-nitrogen stress in trees remains limited. In barley leaves, combined drought and nitrogen deficiency increased the abundance of proteins involved in energy, carbohydrate, and amino acid metabolism, while increased photorespiration and reduced stomatal conductance jointly contributed to lower photosynthetic capacity [34]. In rice, integrated proteomic–metabolomic profiling showed that nitrogen management before drought enhanced NR and GS activities, promoted osmolyte accumulation, and improved yield relative to nitrogen application after drought [81]. However, these findings derive from herbaceous annual crops and may not directly reflect responses in woody perennials. Proteomics can determine whether transcriptional changes are accompanied by changes in protein abundance, but it cannot by itself establish enzyme activity, metabolic flux, or causal relationships. Accordingly, tree studies should integrate proteomic analyses with gas-exchange measurements, nitrogen-uptake assays, enzyme activity measurements, and factorial models that explicitly test drought × nitrogen interactions.

Metabolomics provides a complementary, phenotype-proximal layer by quantifying soluble sugars, amino acids, organic acids, and secondary metabolites. Metabolomic studies in forest trees have expanded across *Pinus*, *Quercus*, and *Eucalyptus* species [12], although metabolite profiles are strongly influenced by tissue type, season, developmental stage, and analytical platform. Findings from combined heat and drought experiments in *P. massoniana* [67], together with studies of herbaceous systems exposed to drought, nitrogen limitation, or other stress combinations [70,82,83], indicate that carbon- and nitrogen-related metabolites are responsive to environmental stress. However, these findings do not yet establish a conserved metabolic signature of combined drought and low-nitrogen stress in trees. Targeted isotope tracing, combined with matched transcriptomic, proteomic, and metabolomic sampling, is therefore needed to distinguish changes in metabolite pool sizes from alterations in metabolic flux.

Epigenomic approaches can identify changes in DNA methylation, histone modifications, and RNA modifications associated with stress memory. In the stem-differentiating xylem of *P. trichocarpa*, drought increased Nm6A modification levels in cellulose- and lignin-related transcripts while weakening transcriptome-proteome coupling, providing evidence for an epitranscriptomic layer of drought regulation during wood formation [84]. However, interpretation is constrained by cell-type heterogeneity and the difficulty of distinguishing stable stress memory from transient responses. Current literature remains largely methodological [85], and direct evidence for epigenetic regulation under factorial drought × low-nitrogen stress in trees is limited. Longitudinal, cell-type-resolved sampling combined with functional perturbation will be necessary to determine whether specific epigenomic marks play causal roles in responses to combined stress.

### 6.3. CRISPR/Cas9 Functional Validation

CRISPR/Cas9 has enabled functional validation of individual drought- and nitrogen-related genes in trees, with most studies conducted in *Populus*. The evidence should be interpreted according to the specific stress context in which each phenotype was evaluated. Drought-related targets include PdNF-YB21, PagEPFL9, PagHB7, PagHCF106, and PagHyPRP1, for which CRISPR/Cas9-mediated editing produced phenotypes involving root architecture, xylem development, stomatal density, or stomatal aperture under drought-related conditions [13,86,87,88]. Beyond *Populus*, a growth-point transformation-based CRISPR/Cas9 system in *Fraxinus mandshurica* was used to validate FmbHLH1 as a positive regulator of drought tolerance, acting through ROS scavenging and osmotic adjustment [89]. Second, the GATA transcription factor GNC has been functionally validated as a positive regulator of nitrate uptake, nitrogen assimilation, growth, and nitrogen-use efficiency (NUE). In poplar, *gnc* knockouts exhibit reduced nitrate acquisition and slower growth, whereas GNC overexpression directly upregulates *PdNRT2.4b*, *PdNR*, *PdNiR*, and *PdGS2* and improves NUE, particularly under low-nitrate conditions [57]. Third, GNC represents a candidate dual-function regulator because an independent study in poplar linked it to drought tolerance. PdGNC directly activates PdHXK1, promoting guard-cell NO and H_2_O_2_ production and reducing stomatal aperture, whereas *gnc* mutants exhibit greater water loss and increased drought sensitivity [58]. However, these independent assays do not constitute functional validation of a combined drought × low-nitrogen phenotype (Table 3).

To date, no tree study has reported CRISPR/Cas9-based functional testing of a target gene under factorial combined drought and low-nitrogen stress [90]. Combined-stress gene editing is technically feasible in trees, as demonstrated by a CRISPR/Cas9 strategy that simultaneously enhanced drought and salt tolerance in poplar [88]; however, an equivalent factorial drought × low-nitrogen design has not yet been reported. Transcriptomic studies of combined stress in *P. simonii* [24] and *R. pseudoacacia* [63] have identified candidate pathways involving ammonium transporters (AMTs), aspartate-derived amino acid metabolism, and nitrate assimilation that remain to be functionally validated through gene editing. Stable phenotyping of edited poplar also requires careful consideration of chimerism. A second round of shoot regeneration increased the frequency of homozygous mutants [91], while heat treatment combined with adventitious-bud redifferentiation further improved editing efficiency in Shanxin poplar [92]. Recent technological advances have made multiplex editing of candidate genes involved in drought and nitrogen responses increasingly feasible. CRISPR/Cas12a enables multiplexed multi-target knockout in poplar [93], while efficient *Agrobacterium rhizogenes*-mediated transformation combined with transgenic hairy-root shoot regeneration can accelerate the generation of edited poplar lines [94]. Transgene-free ribonucleoprotein delivery has also enabled DNA-free genome editing in poplar meristems [95] and larch [96], extending the RNP-based approach beyond *Populus* to conifers while potentially reducing concerns associated with transgene integration. Combined with factorial phenotyping, explicit tests of drought × nitrogen interactions, and field validation in mature trees, these approaches provide a framework for addressing the current gap in functional validation of combined-stress responses.

## 7. Conclusions

**Established evidence**. Tree studies support the conclusion that drought and nitrogen availability jointly influence photosynthesis, nitrogen uptake, root traits, and carbon allocation. The direction and magnitude of these responses vary with species, genotype, developmental stage, treatment duration, and the form and level of nitrogen supply. Factorial studies and *Populus* functional work provide valuable entry points, but direct genetic validation of an integrated drought and low-nitrogen regulatory module in forest trees remains limited. Crosstalk between ABA and nitrogen transport is therefore best viewed as one prominent integration hypothesis alongside ROS, energy signaling, hydraulic regulation, and transcriptional control.

**Future directions**. Progress requires experiments that separate combined drought and low nitrogen from nitrogen supplementation during drought, quantify interaction terms with factorial designs, and compare species and genotypes using structural, hydraulic, fine-root, physiological, and molecular traits. Single-cell transcriptomics, spatial metabolomics, and epigenomic profiling are promising approaches, but they have not yet established combined-stress mechanisms in mature forest trees. Similarly, multiplex genome editing may test coordinated drought and nitrogen candidates, yet no tree study has directly evaluated edited lines under factorial combined stress. Multi-year experiments and field validation in mature trees are needed before laboratory-derived candidate modules can guide operational breeding or forest management.

Looking ahead, this four-level framework can serve as a unified evaluation scheme, enabling systematic comparison of response patterns across tree species and stress combinations. It can advance the field from fragmented description toward systematic prediction, providing a theoretical foundation for adaptive forest management and molecular breeding of stress-tolerant trees under climate change.

## Figures and Tables

**Table 1:** Expanded comparison of drought and low-nitrogen responses in trees, with implications for factorial combined-stress experiments.

Response Domain	Drought Stress	Low-N Stress	Question Under Combined Stress	Evidence Context
Primary limitation	Water deficit restricts transpiration and CO_2_ supply.	Limited inorganic N restricts N acquisition and photosynthetic protein investment.	Does water limitation intensify N-acquisition constraints beyond the two single effects?	Tree-supported single-stress evidence; factorial testing needed for interaction.
Physiological signaling	ABA-associated regulation of stomata and root water responses.	N-status signaling involving nitrate transport and assimilation pathways.	Which ABA–N transport links persist across tree species and developmental stages?	Mechanistic evidence varies across species and systems.
Photosynthetic carbon assimilation	Stomatal limitation can progress to biochemical limitation as stress intensifies.	Reduced leaf N can constrain chlorophyll and Rubisco investment.	Is the combined limitation additive, synergistic, or genotype dependent?	Requires a drought × N interaction test.
Water-use efficiency	Often changes with stomatal regulation and hydraulic status.	Can change through reduced leaf N, carbon assimilation, and transpiration.	Does WUE increase, decrease, or diverge among species under the combined treatment?	Species- and treatment-dependent.
Root traits and N foraging	Root depth, fine-root turnover, and hydraulic access may be altered.	Fine-root proliferation and transporter regulation can support N foraging.	Can carbon supply sustain root plasticity and N foraging under both constraints?	Direct tree factorial evidence remains limited.
Osmotic and redox adjustment	Osmolyte accumulation and antioxidant responses can accompany dehydration.	N availability can alter amino-acid pools and antioxidant capacity.	Which changes reflect acclimation rather than general stress injury?	Physiological candidates requiring coordinated measurements.
Experimental readout	Water status, gas exchange, hydraulic traits, and root traits.	Leaf N, N uptake, transporter expression, and N-use efficiency.	Full factorial drought × N design with pre-specified interaction contrasts.	Needed to classify additive, synergistic, or antagonistic responses.

*Note:* The table separates well-established single-stress responses from questions that require direct factorial evaluation in trees. The table should not be interpreted as evidence that any listed combined-stress outcome is universal.

**Table 2:** Candidate drought and nitrogen regulatory genes, species context, and evidence strength.

Gene	Species Context	Evidence Category	Supported Interpretation	Reference
*GNC*	*Populus*	II, separate tree drought and N studies	CRISPR-supported nitrogen-use function and separate drought-associated stomatal function, combined-stress phenotype untested	[57,58]
*PuHox52*/*PtrHox52*	*Populus* species	II, complementary ortholog evidence	Nutrient-acquisition and drought-associated functions were reported in separate species and experiments	[54,55]
*GS1a*	*Pinus* transgene in poplar	I, tree single-stress evidence	Supports nitrogen homeostasis maintenance during drought, and the low-nitrogen role requires direct testing	[61]
*SNAC1*, *OsDREB1C*, *GmTDN1*	Rice or crop systems	III, non-tree mechanistic context	Candidate mechanisms for tree testing, not direct forest-tree evidence	[48,49,52,53]

**Table 3:** CRISPR/Cas9 studies in poplar grouped by evidence context for drought and nitrogen traits.

Target Gene	Gene Family	Editing Approach	Phenotype	Reference
*PdNF-YB21*	NF-Y	Knockout	Reduced root growth and drought tolerance; OE enhances drought tolerance	[13]
*PagEPFL9*	EPFL	Knockout	Reduced stomatal density, enhanced drought tolerance	[86]
*PagHB7*	HD-ZIP	Knockout	Increased stomatal density, reduced drought tolerance (negative regulator of EPFL9)	[86]
*PagHCF106*	Chloroplast protein	Promoter editing	Reduced stomatal opening and water loss, elevated guard cell H_2_O_2_	[87]
*GNC*	GATA	Knockout	Reduced NUE and nitrate uptake; OE increases NUE via *NRT2.4b/c* and *NIA1*	[57]
*PagHyPRP1*	Hybrid proline rich protein	Knockout	Reduced ROS accumulation and increased root growth, enhanced drought tolerance	[88]

## Data Availability

No new data were created or analyzed in this study. Data sharing is not applicable to this article.
